# Limited variation in vaccine candidate *Plasmodium falciparum *Merozoite Surface Protein-6 over multiple transmission seasons

**DOI:** 10.1186/1475-2875-9-138

**Published:** 2010-05-24

**Authors:** Aaron T Neal, Stephen J Jordan, Ana L Oliveira, Jean N Hernandez, OraLee H Branch, Julian C Rayner

**Affiliations:** 1 William C Gorgas Center for Geographic Medicine, Division of Infectious Diseases, Department of Medicine, University of Alabama at Birmingham, 845 19th Street South, BBRB 568, Birmingham, AL 35294-2170, USA; 2 Department of Biology, University of Alabama at Birmingham, Birmingham, AL 35294, USA; 3 Department of Cell Biology, University of Alabama at Birmingham, Birmingham, AL 35294, USA; 4 Laboratorio de Investigaciones de Productos Naturales y Antiparasitarios, Universidad Nacional de la Amazonia Peruana, Iquitos, Peru; 5 Department of Medical Parasitology, New York University, 341 East 25th Street, Old Public Health Building Rm 210, 606, New York, NY 10010-2533, USA; 6 Malaria Programme, Wellcome Trust Sanger Institute, Wellcome Trust Genome Campus, Hinxton, Cambridge CB10 1SA, UK

## Abstract

**Background:**

*Plasmodium falciparum *Merozoite Surface Protein-6 (PfMSP6) is a component of the complex proteinacious coat that surrounds *P. falciparum *merozoites. This location, and the presence of anti-PfMSP6 antibodies in *P. falciparum*-exposed individuals, makes PfMSP6 a potential blood stage vaccine target. However, genetic diversity has proven to be a major hurdle for vaccines targeting other blood stage *P. falciparum *antigens, and few endemic field studies assessing *PfMSP6 *gene diversity have been conducted. This study follows *PfMSP6 *diversity in the Peruvian Amazon from 2003 to 2006 and is the first longitudinal assessment of *PfMSP6 *sequence dynamics.

**Methods:**

Parasite DNA was extracted from 506 distinct *P. falciparum *infections spanning the transmission seasons from 2003 to 2006 as part of the Malaria Immunology and Genetics in the Amazon (MIGIA) cohort study near Iquitos, Peru. *PfMSP6 *was amplified from each sample using a nested PCR protocol, genotyped for allele class by agarose gel electrophoresis, and sequenced to detect diversity. Allele frequencies were analysed using JMP v.8.0.1.0 and correlated with clinical and epidemiological data collected as part of the MIGIA project.

**Results:**

Both *PfMSP6 *allele classes, K1-like and 3D7-like, were detected at the study site, confirming that both are globally distributed. Allele frequencies varied significantly between transmission seasons, with 3D7-class alleles dominating and K1-class alleles nearly disappearing in 2005 and 2006. There was a significant association between allele class and village location (p-value = 0.0008), but no statistically significant association between allele class and age, sex, or symptom status. No intra-allele class sequence diversity was detected.

**Conclusions:**

Both *PfMSP6 *allele classes are globally distributed, and this study shows that allele frequencies can fluctuate significantly between communities separated by only a few kilometres, and over time in the same community. By contrast, *PfMSP6 *was highly stable at the sequence level, with no SNPs detected in the 506 samples analysed. This limited diversity supports further investigation of PfMSP6 as a blood stage vaccine candidate, with the clear caveat that any such vaccine must either contain both alleles or generate cross-protective responses that react against both allele classes. Detailed immunoepidemiology studies are needed to establish the viability of these approaches before *PfMSP6 *advances further down the vaccine development pipeline.

## Background

The search for an effective *Plasmodium falciparum *vaccine has been the focus for research efforts by numerous labs over several decades. While the advancement of the pre-erythrocytic vaccine RTS,S, to Phase III trials raises hopes that a vaccine providing some protection against severe malaria could be on the horizon, clear room for improved efficacy remains even within the context of RTS,S [1], making *P. falciparum *vaccine development an ongoing and urgent priority. However, *P. falciparum *presents an overwhelming number of potential vaccine targets, both because its complex life cycle presents several potential stages to target and because the size of the genome presents multiple potential targets at each stage [2]. Given the finite resources available, it is not feasible for every antigen to advance to vaccine trials; there is, therefore, an urgent need for a more rational approach to candidate selection. Such concerns are highlighted by the collaborative Malaria Vaccine Technology Roadmap [3], which proposes that all potential candidates progressing through the vaccine development pipeline be subjected to strict go/no-go criteria; similar issues have been discussed in detail in recent reviews [4,5]. The acquisition of field data describing vaccine candidate sequence diversity and antigenicity in various transmission environments is one key component of these pipeline checkpoints.

*Plasmodium falciparum *Merozoite Surface Protein-6 (PfMSP6) is a potential vaccine candidate at an early stage in development, which still lacks critical field data to inform the go/no-go decisions necessary to either advance it down the pipeline or remove it from consideration. PfMSP6 is a secreted antigen that is proteolytically processed by PfSUB1 into a 36 kDa fragment that associates with fragments of PfMSP1 and PfMSP7 to form a multi-subunit complex on the merozoite surface [6-8]. PfMSP6 is encoded by one gene in a multi-gene family arranged in close proximity along chromosome 10 [9]. All members of this multi-gene family appear to encode merozoite surface antigens, one of which, PfMSP3, has already advanced to several Phase I vaccine trials [10-12]. Although the function of PfMSP6 remains unknown, it has been postulated to participate in erythrocyte recognition and binding, as have many other merozoite surface proteins of unknown functions. PfMSP6 is, therefore, in the right place to be a theoretical vaccine candidate, and its potential is supported by field studies that have observed anti-PfMSP6 antibody responses in serum from *P. falciparum*-infected individuals, which inhibit *P. falciparum *growth *in vitro *[13,14]. However, like many potential vaccine antigens, few detailed genetic or immunoepidemiology studies have been carried out in endemic settings.

Recent studies of two of the most advanced blood stage candidates, PfMSP1 and PfAMA1, have made it clear that sequence diversity is a major hurdle for blood stage vaccines [15], and PfMSP6 is no exception. Past studies of PfMSP6 have shown that like other major merozoite surface antigens PfMSP1 and PfMSP2 [16], it is dimorphic. The two major PfMSP6 allele classes are referred to as K1- and 3D7-like alleles, named for the strains in which they were first identified [17]. Differences between the alleles are largely restricted to a series of indels in the N-terminal domain, but also include single nucleotide polymorphisms (SNPs) within each allele class that are found in both the N-terminal domain preceding the PfSUB1 cleavage site as well as the generally more conserved C-terminal domain [17,18]. A recent study of 89 *PfMSP6 *gene sequences from around the world identified 7 K1-like and 11 3D7-like haplotypes [18].

Although a series of studies have now given a global picture of *PfMSP6 *diversity, no study has assessed how extensively *PfMSP6 *sequences can vary longitudinally at a single study site. To fill this knowledge gap we analysed samples collected between 2003 and 2006 as part of the Malaria Immunology and Genetics in the Amazon (MIGIA) longitudinal cohort study in Zungarococha, a community of four villages located near Iquitos in the Peruvian Amazon. Zungarococha is a hypoendemic transmission environment, with a *P. falciparum *transmission rate of 0.13 infections/person/month during the seven-month transmission season [19]. Consistent with the hypoendemic transmission, *P. falciparum *infections seem to routinely consist of few co-infecting genotypes, but genetic diversity is still easily detectable at the population level with at least five haplotypes defined for *PfMSP1 *Block 2 in this community [20]. The MIGIA project is therefore uniquely suited for vaccine candidate studies, as it allows for the tracking of relatively genetically simple *P. falciparum *infections that are widely spaced temporally but with considerable genetic diversity at the population level. Furthermore, since samples are collected as part of an ongoing longitudinal study, genotype data can be correlated with detailed clinical and epidemiological data. To further clarify the potential of PfMSP6 as a vaccine candidate, *PfMSP6 *sequence diversity was characterized in 506 *P. falciparum *samples collected between 2003 and 2006 as part of the MIGIA cohort, and the resulting genotypes were compared with clinical and epidemiological data. The results inform the rational assessment of PfMSP6 as a vaccine candidate.

## Methods

### Study site

A complete description of the MIGIA longitudinal cohort study has been published previously [19]. In brief, the study site consists of four villages that comprise the Zungarococha community: Zungarococha village, Puerto Almendra, Ninarumi, and Llanchama. The community is located in a stable hypoendemic malaria transmission environment. Since 1994, both *P. vivax *and *P. falciparum *have been transmitted during the annual seven-month malaria season between January and July, with the primary vector being *Anopheles darlingi *[21]. Community residents have equal access to healthcare in the community health centre staffed by MIGIA cohort physicians, live in similar housing conditions, and have similar income levels. Travel outside of the community is rare, as women typically work in or near their home and men work in local agriculture or as fishermen along the nearby Nanay River, a tributary of the Amazon River. Travel that does occur is frequently to Iquitos, a city free of malaria transmission.

### Blood collection and DNA extraction

The sample collection process, involving both passive and active case detection, has been detailed previously [19]. Briefly, passive case detection occurs when symptomatic individuals seek care at the community health outpost, where confirmation of malaria is made by microscopy. In contrast, active case detection occurs through routine community visits and identifies asymptomatic individuals. This study design increases the likelihood of sampling both symptomatic and asymptomatic *P. falciparum *infections. All patients submit a 0.5 ml blood sample and, upon malaria diagnosis are re-evaluated, submit another blood sample, and are cleared of parasites by co-administration of mefloquine and artesunate. Samples are separated by centrifugation into serum and packed erythrocyte fractions. *Plasmodium *DNA is extracted from the erythrocyte fraction using a Blood DNA kit (Qiagen), and the species is identified by PCR using species-specific primers. All samples are catalogued and stored at -80°C until needed. For this study, *P. falciparum *isolates collected between from 2003 to 2006 were selected at random, excluding only subsequent infections in the same individual that occurred within 60 days of the initial infection in order to reduce the risk of duplication due to parasite recrudescence.

### Nested PCR and genotyping

The region of *PfMSP6 *where all detected inter- and intra-allele genetic diversity has been shown to occur was amplified using a nested PCR protocol with the external primers 5'--CGTGAATACTATTTTCGTTACTT--3' and 5'--CAGCAGTCTTTTTTGTTTCAT--3' and the internal primers 5'--CCCCATCAATCTTATGTCCAG--3' and 5'--CACTTTCTTCATCTATGTCATCTTCTT--3'. The amplified fragment corresponds to nucleotides 221-784 of the reference 3D7 *PfMSP6 *sequence, excluding primer sequences. 1.0 μl of genomic DNA, extracted from *P. falciparum*-infected patients, was amplified using ChoiceTaq (Denville) in 35 cycles of 95°C for 30 seconds, 51.1°C for 30 seconds, 65°C for 1 minute, and 65°C for 5 minutes. For the nested PCR reaction, all conditions remained the same except that 1 μl of the primary PCR reaction was used as the template. Multiple negative controls were included in each PCR experiment to monitor for contamination. Allele-typing of *PfMSP6 *was performed using ethidium bromide-stained agarose gel electrophoresis. *PfMSP6 *amplified from the *P. falciparum *strains HB3 and Dd2 were used as controls and run on all agarose gels to aid classification of infections as either 3D7-like or K1-like allele type. All PCR products were subsequently sequenced using sequencing primer 5'--CTTCTTCATTTTCTTCTATCTC--3'. Sequence alignment was performed using CodonCode Aligner 3.0.3 (CodonCode Corporation).

### Statistical analysis

Descriptive statistics, such as allele frequencies, percentages, and means, were used to quantitatively summarize all data sets. Comparisons between allele classes for age group, sex, communities, symptom status, subsequent allele, subsequent *P. falciparum *infections, year of infection, time to next *P. falciparum *infection, and comparisons between all other variables of interest were performed using Pearson chi-square or Fisher's exact chi-square, when necessary. Infections were classified as symptomatic if patients experienced febrile illness at least two days prior to diagnosis, had a detected fever ≥ 38.3°C, and/or had a packed cell haematocrit < 30%. Comparisons between means of actual age and days to next *P. falciparum *infection were performed using the independent t-test. All statistical tests were two-tailed and performed using a 5% significance level in JMP (version 8.0.1.0; SAS Institute, Inc., Cary, NC).

### Ethical approval

This study was approved by the Institutional Review Boards of the University of Alabama at Birmingham, New York University and the Peruvian Ministerio de Salud, Instituto Naccional de Salud. All participants in the study gave informed consent in writing prior to enrollment in the study.

## Results

### Significant variation in *PfMSP6 *allele frequency is observed across transmission seasons

506 samples were selected from *P. falciparum *infections detected in the MIGIA cohort over the 2003-2006 transmission seasons. Infections that occurred in a given individual within 60 days of a prior infection were excluded. This, combined with the fact that all patients were treated upon detection of a *P. falciparum *infection (see Methods), significantly reduces the likelihood that any given infection was represented more than once in the sample set.

**Figure 1:**
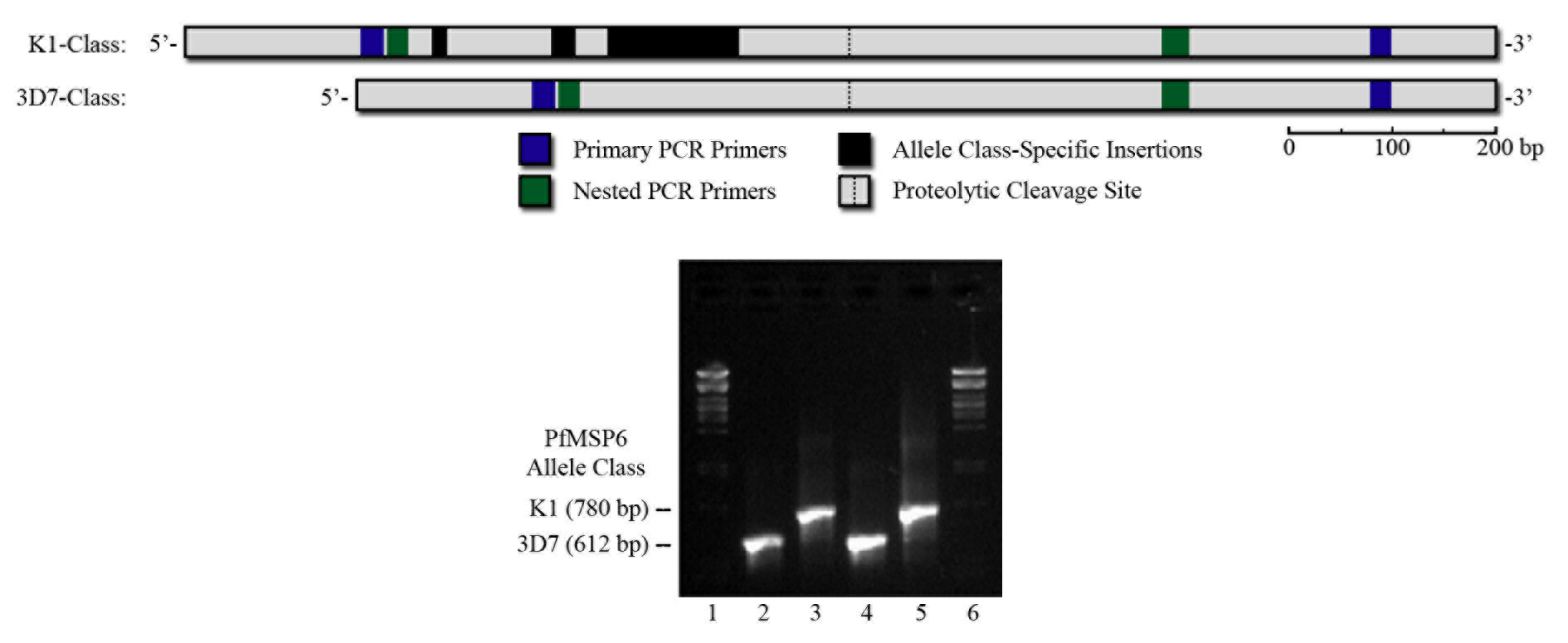
**Genotyping *PfMSP6 *using nested PCR and agarose gel electrophoresis**. This study utilizes a nested PCR protocol to amplify the region of *PfMSP6 *where most inter- and intra-allele genetic diversity has been shown to occur. The two major *PfMSP6 *allele types, K1-class and 3D7-class alleles, result in nested PCR products of significantly different sizes, and allele genotyping was scored using agarose gel electrophoresis, as shown.

**Figure 2:**
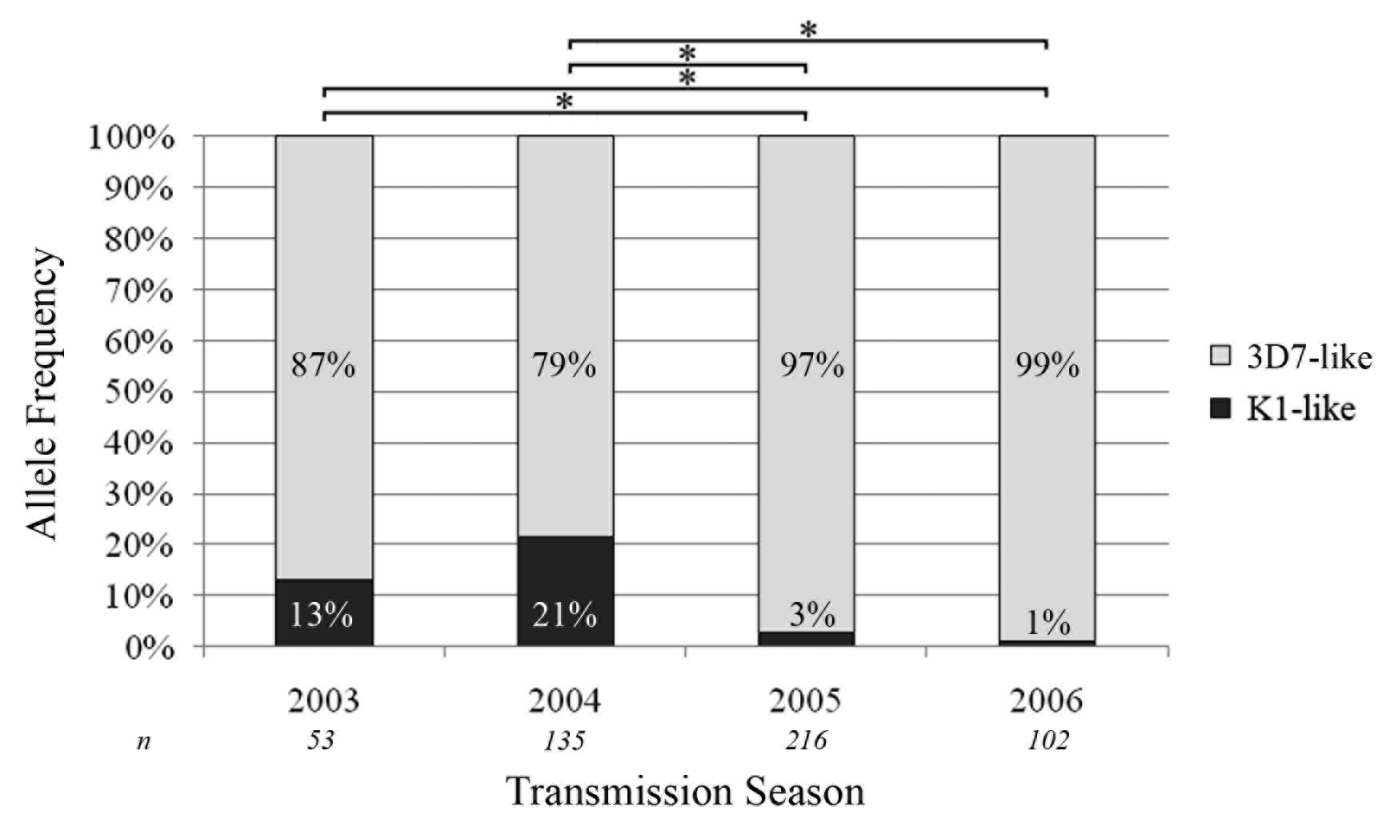
***PfMSP6 *allele distribution changes significantly over consecutive transmission seasons**. Distribution of the K1- and 3D7-like allele frequencies across the 2003-2006 transmission seasons showed a significant decline in K1-allele frequency between 2003/2004 and 2005/2006. Each bar represents the percentage of each allele type detected in *n *samples for the given year, and * denotes significant differences between paired years with *p *= 0.0001 in χ^2 ^analysis.

Nested PCR was used to amplify nucleotides 221-784 of *PfMSP6 *from each sample (nucleotide position taken from the reference 3D7 *PfMSP6 *sequence; Figure 1), a fragment that includes both the known dimorphic regions as well as previously identified SNPs both before and after the PfSUB1 cleavage site [17]. Samples were allele-typed by agarose gel electrophoresis; of the 506 samples genotyped, 463 contained the 3D7 *PfMSP6 *allele-class (91.5%) and 43 contained the K1 allele-class (8.5%). This supports previous reports that 3D7-like alleles are more prevalent world-wide (73.8% of published *PfMSP6 *sequences, [18]). No mixed infections of K1- and 3D7-class alleles were detected. The observed allele frequency varied across the transmission seasons, with the frequency of K1-class infections decreasing from 21.5% in 2004 to 1.0% in 2006, and with allele frequencies in 2003 and 2004 statistically significantly different to those in 2005 and 2006 (Figure 2).

### Correlation of *PfMSP6 *allele frequencies with epidemiological and clinical data

**Figure 3:**
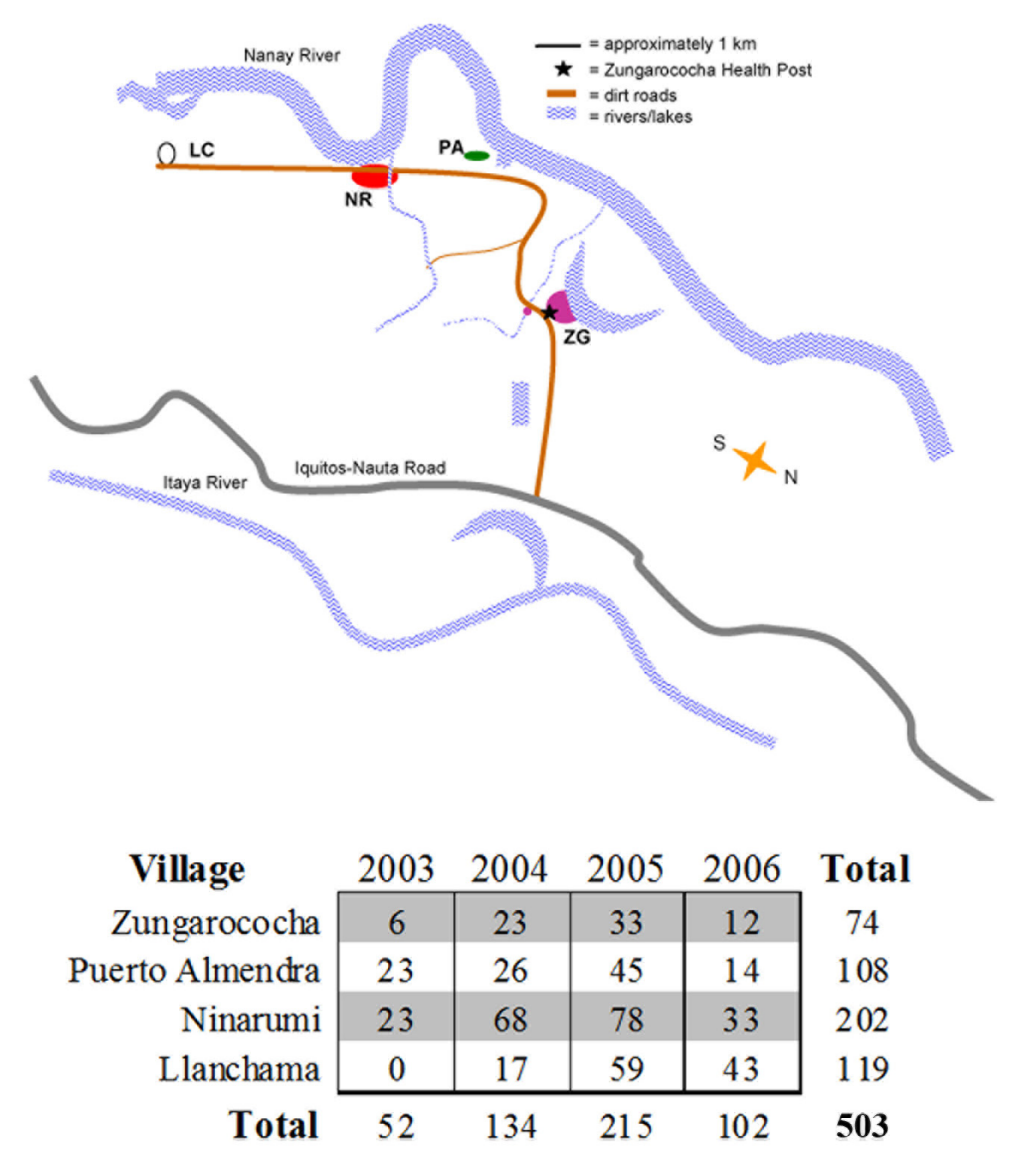
**Distribution of *P. falciparum *samples analysed**. Zungarococha is a small community in the Peruvian Amazon near Iquitos consisting of four separate villages of varying size: Zungarococha village (population = 805), Puerto Almendra (population = 272), Ninarumi (population = 590), and Llanchama (population = 203). Since 2003, the MIGIA cohort study has monitored *P. falciparum *transmission throughout the community using both active and passive sample detection (for details, see Methods section). Samples were collected from all four villages; the number genotyped from each village reflects variation in both the size of the villages and the burden of *P. falciparum *infection.

The MIGIA cohort study results in the collection of wide-ranging epidemiological and clinical data [19]. *PfMSP6 *allele frequency was compared with this data, in order to establish whether certain alleles associated with specific epidemiological features. While there were no significant associations between *PfMSP6 *allele frequency and age or gender, there was a significant association between *PfMSP6 *allele frequency and community (Table 1). The Zungarococha community consists of four independent villages (map shown in Figure 3), and *P. falciparum *infection burden is not uniform across the villages: Zungarococha village, the largest in the community, carries the smallest burden of infection. Village location data was available for 503 samples (Figure 3); comparing allele frequencies with location revealed that Puerto Almendra had a significant increase in K1-class infections (> 2-fold, *p *= 0.0061) compared to the other villages, while Llanchama had a significant decrease in K1-class infections (> 14-fold, *p *= 0.0007) compared to the other villages (Table 2).

**Table 1:** Association of *PfMSP6 *allele type with MIGIA cohort study epidemiological data.

Factor	3D7-Class	K1-Class	*P*-value
**Age Group**			0.7530
< 15 years	148	15	
≥ 15 years	307	28	

**Gender**			0.7276
Male	250	24	
Female	210	18	

**Community**			0.0008*
Zungarococha	70	4	
Puerto Almendra	92	16	
Ninarumi	181	21	
Llanchama	118	1	

**Symptom Status**			0.3757
Asymptomatic	118	12	
Symptomatic	327	24	

Using χ^2 ^analysis, relationships between *PfMSP6 *allele class and various epidemiological factors were assessed; statistically significant associations are indicated with an asterisk. There was a significant association between the infecting allele type and the village at which the *P. falciparum *samples were collected.

**Table 2:** Association of *PfMSP6 *allele type with village of residence.

Village	3D7-Class	K1-Class	*P*-value
**Zungarococha**			0.3215
ZG	70	4	
Others	391	38	

**Puerto Almendra**			0.0061*
PA	92	16	
Others	369	26	

**Ninarumi**			0.1742
NR	181	21	
Others	280	21	

**Llanchama**			0.0007*
LL	118	1	
Others	343	41	

Using χ^2 ^analysis, relationships between infecting *PfMSP6 *allele and village of residence were assessed; statistically significant associations are indicated with an asterisk. Puerto Almendra showed a statistically significant increase in K1 infections (>2-fold) compared to other villages. Llanchama showed a statistically significant decrease in K1 infections (>14-fold) compared to other villages.

Comparison with clinical data revealed no association between *PfMSP6 *allele class and symptom status (Table 1). The longitudinal nature of the MIGIA cohort study, where individuals are followed over a long period of time, also allows the analysis of subsequent infections in the same individuals spaced by at least 60 days. Of the 506 infections genotyped, 79 infections were identified as successive infection pairs, where individuals had two distinct *P. falciparum *infections within < 500 days. Of these 79 infections, 65 were successfully genotyped and 53 had comparable clinical symptom data. No statistically significant associations between subsequent allele, subsequent *P. falciparum *infection, or time to next infection were detected (Table 3). If allele-specific immunity exists against PfMSP6, then a subsequent infection might be expected to be of a different allele-class than the original infection. Comparing the allele types present in the initial and subsequent infections yielded no significant departures from the average allele frequencies, but this analysis is not powered to detect significant associations because of the low number of K1 infections at the study site, such that of the individuals with successive infection pairs, only one had a K1-class infection at their initial visit, and only one individual had a K1-class infection during the subsequent infection.

**Table 3:** Association of *PfMSP6 *allele with subsequent infection data.

	3D7-Class	K1-Class	*P*-value
**Subsequent Allele**			0.3432
3D7-Class	55	6	
K1-Class	3	1	

**Subsequent Pf Infection**			0.5720
No	392	35	
Yes	71	8	

**Next Infection (days)**			0.6503
Mean	318	276	
n	61	4	

Using χ^2 ^analysis, relationships between initial *PfMSP6 *infection with subsequent *P. falciparum *infection and subsequent *PfMSP6 *allele type were assessed. Time between initial and subsequent infections was assessed using a t-test; no statistically significant associations were detected.

### Sequence variation in *PfMSP6 *is limited

To investigate intra-allele sequence diversity, all 506 samples were sequenced and compared to published sequences. Any samples that showed potential SNPs were re-amplified and re-sequenced to eliminate the possibility of PCR-induced error. All 3D7-class *PfMSP6 *alleles were identical to the HB3 strain [GenBank:AY518889], and all K1-class alleles were identical to the K1 strain [GenBank:AY518890]; there was no sequence level diversity across all four transmission seasons.

This sequence stability is in contrast to the adjacent gene on chromosome 10, *PfMSP3*, which also encodes a related merozoite surface antigen that is under active development as a vaccine candidate [10-12]. Although *PfMSP3 *sequence diversity at this study site was also limited, previous studies at the same site did reveal rare *PfMSP3 *sequence variation, all of which shared the same single SNP [22]. Of the 506 samples genotyped for *PfMSP6 *in this study, 10 had previously been shown to have *PfMSP3 *SNPs; none of these 10 samples contained *PfMSP6 *sequence variants.

Comparison of *PfMSP3 *and *PfMSP6 *gentoypes revealed some evidence of recombination between these two adjacent genes. *PfMSP3 *also consists of two defined allele classes, 3D7-like and K1-like [23,24]. The majority of samples genotyped in both studies contained either both 3D7-class *PfMSP3 *and 3D7-class *PfMSP6 *alleles, or K1-class *PfMSP3 *and K1-class *PfMSP6 *alleles. Only five samples had mixed *PfMSP3 *and *PfMSP6 *alleles, in keeping with established low *P. falciparum *recombination rates in South America [25]: three infections where a K1-class *PfMSP6 *allele was paired with a 3D7-class *PfMSP3 *allele, and two infections where a 3D7-class *PfMSP6 *allele was paired with a K1-class *PfMSP3 *allele.

## Discussion

Recent discussions of global malaria elimination as a stated goal for the research community have increased the focus on the decades-long hunt for an effective *P. falciparum *vaccine [26]. While it is sometimes debated whether a vaccine is a necessary constituent of such campaigns, or precisely which stage should be targeted [27], the possible emergence of *P. falciparum *strains resistant to artemisinin, the current front-line drug in global malaria treatment campaigns [28], emphasizes that it may be premature to reject any approach if the global effort against malaria is to be successful.

While a vaccine targeting a sporozoite stage antigen is currently undergoing Phase III trials [1], vaccines targeting asexual stage antigens, which in theory would have the clinical advantage of limiting symptoms even if they were not completely effective in eliminating parasites, have lagged somewhat in development. Genetic diversity is clearly a major hurdle for many asexual antigen vaccines [14], and is presumably responsible for the disappointing results from recent field trials of a PfMSP1-based vaccine [29]. To avoid similar disappointment in the future, it is essential that all potential vaccine candidates undergo rigorous go/no-go analysis in the pre-clinical phase, with candidates being eliminated from consideration if they do not meet certain criteria. Several approaches can be used to inform these go/no-go decisions, including experimental genetic manipulation and detailed field studies investigating both natural genetic diversity and immunoepidemiology.

PfMSP6 is a merozoite candidate antigen at an early stage of pre-clinical development, lacking significant field data that supports its potential role as a viable vaccine candidate. To help inform go/no-go decisions for PfMSP6-based vaccine development, *PfMSP6 *diversity was followed over multiple transmission seasons in a hypoendemic transmission environment in Peru. At a sequence level, *PfMSP6 *diversity was very limited in this setting. No intra-allele sequence variants were found in over 500 distinct *P. falciparum *infections spanning four transmission seasons at the MIGIA cohort study site near Iquitos, Peru. While *P. falciparum *genetic diversity is, in general, much lower in South America than other regions [25], SNPs were detected in two other vaccine antigens, *PfMSP1*_*19 *_and *PfMSP3*, at the same site over the same period [20,22]. Genetic stability in low transmission is a generally low bar for vaccine candidate antigens, but the relative stability of the *PfMSP6 *gene compared to other vaccine antigens even in this setting certainly supports its further investigation as a vaccine candidate.

However, although *PfMSP6 *was stable at a sequence level, the frequency of the two *PfMSP6 *allele classes fluctuated significantly between transmission seasons and between villages within the study site. K1 allele-class infections, which are in the minority at this study site just as they appear to be globally [18], exhibited an overall downward trend over the study period, from 13.2% of total infections in 2003 to 1.0% of total infections in 2006, with a statistically significant drop-off in infections between 2003/2004 and 2005/2006. Within the Zungarococha community, K1-class infections were over-represented in Puerto Almendra (*p *= 0.0061) and under-represented in Llanchama (*p *= 0.0007), despite the fact that the two villages are less than 3 km apart. Population differences between the two villages provide a potential explanation. Puerto Almendra is a heavy-traffic village on the Nanay River, and the associated activities of non-residents and position on the riverbank may increase the likelihood of introduction of new allele types through infectious travellers or transport of infected mosquitoes from upstream transmission. Llanchama, by contrast, is the smallest and most isolated village in the community, with little exposure to outside infections.

In addition to measuring *PfMSP6 *allele-class diversity at the community level, clinical and epidemiological data collected as part of the MIGIA cohort study allowed for the assessment of specific *PfMSP6 *allele classes with clinical data, which revealed no association of allele class with any clinical data or with any other epidemiological data. The extensive longitudinal data collected as part of the MIGIA study also allowed testing for evidence of allele-specific immunity by assessing whether either infecting allele-class correlated with an increased frequency of subsequent infections, the length of time until subsequent infection, the allele-class of subsequent infections, or an increased frequency of asymptomatic subsequent infections. No significant associations were observed, but the possibility of allele-specific host immune responses should not be excluded due to the lack of statistical power from the infrequent number of infections characteristic of a hypoendemic environment, and the low incidence of K1-class infections in particular.

While the absence of intra-allele class sequence variation in *PfMSP6 *is a positive attribute for any vaccine candidate, the dynamism of *PfMSP6 *allele frequencies even in a hypoendemic transmission environment such as the one in the MIGIA cohort emphasizes the fact that any PfMSP6-based vaccine must be able to provide protection against both allele classes. This could be achieved by using a fragment that is conserved between both alleles, or a fragment that is not conserved but is able to induce cross-protection, or by mixing antigens from both allele classes. The simplest way to distinguish between the viability of these approaches it to use immunoepidemiology studies to establish which domains of PfMSP6 are immunogenic in natural *P. falciparum *infections, and whether the antibodies raised against them are able to cross-react between allele classes.

## Conclusions

PfMSP6 is a *P. falciparum *asexual vaccine candidate with limited pre-clinical data to support its advancement or elimination from further development. Data from *P. falciparum *infections in the Peruvian Amazon establishes that it is significantly less genetically variable than other merozoite surface vaccine candidate antigens at this site, but *PfMSP6 *allele frequencies can vary significantly both over time and between local villages. The design of any future PfMSP6-based vaccine must take this data into account and provide protection against both allele classes if it is to warrant further development.

## Competing interests

The authors declare that they have no competing interests.

## Authors' contributions

ATN carried out the sample and data analysis, assisted by SJJ and ALO. OLB established and directed the MIGIA project and provided all samples and epidemiologic data for analysis. JNH managed the cohort study and led the collection of clinical and epidemiological data. ATN, SJJ, ALO, OLB and JCR wrote the manuscript. JCR conceived of the study and supervised all experiments and analysis. All authors read and approved the final manuscript.
